# Prenatal Alcohol Exposure and the Developing Immune System

**DOI:** 10.35946/arcr.v37.2.11

**Published:** 2015

**Authors:** Theresa W. Gauthier

**Affiliations:** Theresa W. Gauthier, M.D., is an associate professor in the Department of Pediatrics at Emory University School of Medicine, Atlanta, Georgia.

**Keywords:** Alcohol in utero, prenatal alcohol exposure, fetal alcohol effects, alcohol-related intrauterine disorder, fetal alcohol syndrome, fetal alcohol spectrum disorders, immune system, immune function, fetal development, prenatal development, pregnancy, premature birth

## Abstract

Evidence from research in humans and animals suggest that ingesting alcohol during pregnancy can disrupt the fetal immune system and result in an increased risk of infections and disease in newborns that may persist throughout life. Alcohol may have indirect effects on the immune system by increasing the risk of premature birth, which itself is a risk factor for immune-related problems. Animal studies suggest that alcohol exposure directly disrupts the developing immune system. A comprehensive knowledge of the mechanisms underlying alcohol’s effects on the developing immune system only will become clear once researchers establish improved methods for identifying newborns exposed to alcohol in utero.

Most Americans are aware that drinking alcohol during pregnancy can injure the developing fetus. Fetal alcohol syndrome (FAS) and fetal alcohol spectrum disorders (FASD), with their developmental, cognitive, and behavioral consequences, probably are the best known dangers (Bakoyiannis et al. 2014; Centers for Disease Control and Prevention [CDC] 2009). However, drinking during pregnancy also can disrupt other areas of fetal development besides the brain, including the developing immune system. Studies in humans and animals suggest that alcohol does, in fact, affect the developing immune system and leads to increased risk of infection and disease in infants exposed to alcohol in utero.

Alcohol’s effect on the developing immune system is apparent in infants born at term gestation, with studies showing that these babies are at increased risk of infection when exposed to alcohol in utero. However, premature infants are at even higher risk of infection for multiple reasons. For one, in utero alcohol exposure is associated with premature birth, which independently increases immune-related risks. In addition, animal studies show that alcohol has a direct effect on specific aspects of immune function, particularly in the developing lung. This article will discuss the short and long-term effects of drinking during pregnancy on the immune system of the developing fetus (see the figure for an overview).

Understanding the full extent of alcohol’s threat to the developing fetus is critical because, despite increased awareness about the risks of drinking during pregnancy, a significant number of women continue to do so. Based on a large household survey, the CDC estimates that 1 in 13 women drink alcohol during pregnancy (CDC 2012). Studies interviewing women just after birth have found that between 25 and 35 percent of newborns were exposed to alcohol in utero (Gauthier et al. 2005*a*; Lester et al. 2001). Interestingly, and contrary to many traditional biases (Goldberg 1995; Hans 1999), these studies also found that older women and women of higher socioeconomic status were as or more likely to drink during pregnancy than younger, less affluent women (CDC 2012; Gauthier 2005*a*; Hutchinson et al. 2013). Because most studies of maternal alcohol use rely on self-reports, and there remains significant stigma associated with alcohol use during pregnancy, these findings likely underestimate the true extent of this problem.

## Risk of Alcohol Exposure in Term Infants

Although full-term babies generally are healthier compared with babies born prematurely, there is some evidence that maternal alcohol exposure can increase the risk of neonatal infection even in term newborns. One study, for example, evaluated neonatal infections in 872 newborns with gestational age greater than or equal to 36 weeks. Infants whose mothers reported any alcohol use, excessive drinking, or smoking during pregnancy were more likely to have an infection than infants whose mothers reported that they abstained from alcohol ingestion or cigarette smoking (Gauthier et al. 2005*a*). When the researchers controlled for race and smoking, infants that were small for gestational age (SGA) and whose mothers used any alcohol had a 2.5-fold increased risk of infection. Excessive alcohol use by the mother in these SGA infants increased the risk of infection three- to fourfold. Even after controlling for low maternal income, smoking, and having a baby that was SGA, the researchers found that the newborns were three times more likely to have a neonatal infection if their mothers drank more than seven drinks per week during pregnancy (Gauthier et al. 2005*a*). This effect was most significant if the alcohol use occurred in the second trimester of pregnancy, a time when the neonatal immune system is developing. These findings suggest that maternal alcohol ingestion may increase the risk of potentially serious acute health problems in the postnatal period, even in full-term infants. Risks of alcohol exposure are even more significant for those babies born prematurely. We will therefore focus the remainder of the article on this uniquely vulnerable population.

## Alcohol’s Link to Premature Birth

Premature infants are at increased risk for a variety of significant medical complications, including respiratory, cardiac, neurological, and gastrointestinal problems as well as infection and infection-related complications. Alcohol consumed during pregnancy, researchers postulate, may exacerbate these problems. In addition, research continues to evaluate the hypothesis that drinking during pregnancy can independently increase the risk of premature birth.

The strength of the potential link between alcohol and premature birth remains under debate, because several studies have failed to demonstrate a significant relationship between alcohol and prematurity (Bailey and Sokol 2008). However, Bailey and Sokol (2008) argue that the suspected link is strengthened if they account for potential flaws in study design, particularly among women who drink heavily or binge drink during pregnancy (Bailey and Sokol 2011). In fact, the data thus far do not demonstrate a link between low-to-moderate drinking during pregnancy and the risk of premature delivery (Bailey and Sokol 2011), but multiple studies demonstrate a two- to threefold increase in the risk of premature delivery for women who drink heavily or binge drink during pregnancy (Kesmodel et al. 2000; Mullally et al. 2011; O’Leary et al. 2009; Sokol et al. 2007). Further-more, heavy drinkers exhibited a dramatic 35-fold increased risk of delivering their babies extremely prematurely (earlier than 32 weeks) compared with women who did not drink during pregnancy (Mullally et al. 2011; Sokol et al. 2007). Therefore, some authors propose that extreme prematurity is an alcohol-related birth defect (Sokol et al. 2007).

Maternal alcohol use also has been associated with multiple risk factors that independently increase the risk of premature delivery. For example, chorioamnionitis—an inflammation of the fetal membranes due to a bacterial infection—confers a significant risk for preterm labor and premature delivery and also increases the risk of multiple adverse outcomes for premature newborns (Pappas et al. 2014). In multiple reviews, maternal alcohol use significantly increased the risk of chorioamnionitis, with risks ranging from five to more than seven times higher when compared with pregnancies without alcohol exposure (de Wit et al. 2013; Hutchinson et al. 2013). Placental abruption, a dangerous condition when the placental lining separates from the uterus, also increases the risk of premature delivery (Sokol et al. 2007). A large review of risk factors for placental abruption suggested that maternal alcohol ingestion increased the risk of abruption by more than twofold (Martinelli et al. 2012).

Although these findings suggest that maternal alcohol use is a risk factor for premature delivery, identification of alcohol-exposed term and premature newborns using traditional clinical tools is poor in both the well-baby nursery as well as newborn intensive care units (Little et al. 1990; Stoler and Holmes 1999). Given this, in order to accurately determine alcohol’s adverse effects on premature newborns, it is paramount to validate biomarkers of alcohol exposure in this already at-risk population. One potential marker is a product of alcohol metabolism called fatty acid ethyl esters, which studies suggest accurately determine alcohol exposure in term newborns and in adults (Bearer et al. 1992, 2005; Best and Laposata 2003; Kulaga et al. 2006; Laposata and Lange 1986). Additional research examining ways to improve the accuracy of identifying alcohol-exposed newborns has evaluated the combination of other products of nonoxidative ethanol metabolism including phosphatidylethanol (PEth), ethyl glucuronide (EtG), and ethyl sulfate (EtS) (Bakhireva et al. 2014; Joya et al. 2012). To date, researchers have investigated these methods only in term pregnancies. They now need to test them in premature newborns exposed to alcohol. Once there is an accurate, safe, and convenient way to identify premature newborns exposed to alcohol, it will enable researchers to determine how prenatal alcohol exposure contributes to the development of common disorders faced by the premature population, including late-onset sepsis (infection), the lung condition bronchopulmonary dysplasia, the gastrointestinal disease necrotizing enterocolitis, and neurodevelopmental delays.

## Premature Birth and the Risk of Infection

Despite a lack of biomarkers to specifically identify alcohol-exposed premature infants, research can begin to indirectly link in utero alcohol exposure to increased risk of infections and infection-related illnesses in this population. For all newborns, but particularly those born prematurely, infections play a significant role in illness and mortality (Alarcon et al. 2004; Benjamin et al. 2006; Cordero et al. 2004; Stoll et al. 2010). Even with antibiotic therapy and modern neonatal intensive care, the risk of bacterial infections remains disproportionately elevated in premature newborns and those born within minority groups (Stoll et al. 1998, 2002). Bacterial infection in the premature population increases the risk of a variety of complications including patent ductus arteriosus, in which abnormal blood flow persists between the pulmonary artery and the aorta; necrotizing enterocolitis, in which intestinal tissue becomes diseased and can die; bronchopulmonary dysplasia, a chronic and serious lung condition (Stoll et al. 2002); and neurodevelopmental delays (Adams-Chapman and Stoll 2006; Stoll et al. 2004).

Even as the premature newborn grows, it remains at increased risk for significant problems related to respiratory infections, particularly those of viral origin. Although immunization strategies such as Palivizumab, which aims to prevent serious and often life-threatening lung infections caused by respiratory syncytial virus (RSV), target premature newborns and at-risk newborns with significant lung disease, the growing premature newborn remains at an increased risk for RSV infection, particularly in the lower respiratory tract of the lung (American Academy of Pediatrics 2009; Hall et al. 2009). Furthermore, children born prematurely continue to be at increased risk for severe influenza infections, which adversely affect their long term prognosis (Izurieta et al. 2000; Louie et al. 2006).

Data directly linking in utero alcohol exposure to infections in infants and children are sparse, but some studies suggest an increased risk of neonatal bacterial infection. For example, a small study of children diagnosed with FAS found abnormal lymphocytes and increased rates of bacterial infections such as meningitis, pneumonia, and otitis (Johnson et al. 1981). In addition, hospital stays during the first year of life are approximately three times longer for infants with FAS compared with matched control infants (12.1 days vs. 3.9 days, respectively), with pneumonia being one of the main reasons for hospitalization (Kvigne et al. 2009). Drugs, including alcohol, also potentially increase the risk of maternal to fetal HIV transmission. There is a well-described association between alcohol abuse, the use of other drugs of abuse, and the acquisition and progression of HIV/AIDS among women (Wang and Ho 2011; also see the article by Bagby and colleagues).

The question remains, however, whether alcohol exacerbates the increased risk of infection already occurring in premature infants. To test this, we performed a small case-control analysis of very-low-birth-weight, premature newborns (birth weight less than 1,500 grams). We used social-work interviews to assess maternal alcohol use during pregnancy and found that premature babies exposed to alcohol in utero were 15 times more likely to show signs of early-onset bacterial sepsis than matched premature newborns without in utero alcohol exposure. This risk of early-onset bacterial sepsis with alcohol exposure remained even after we controlled for chorioamnionitis and premature prolonged rupture of membranes (Gauthier 2004). This study suggests that maternal alcohol use during pregnancy increases the risk of infection in the premature newborn, but much investigation still is necessary to fully define the influence of maternal alcohol use on neonatal infection.

Animal models of fetal ethanol exposure play an important role in furthering this research. These models help identify mechanisms underlying alcohol’s detrimental effects on immune defense (Gauthier et al. 2005*b*, 2010; Lazic et al. 2007; McGill et al. 2009; Sozo et al. 2009), and they not only support these early clinical findings but also suggest that in utero exposure alters multiple arms of innate immunity in the developing fetal lung, as we discuss below.

## Maternal Alcohol Ingestion and Lung Immunity

As mentioned above, viral-mediated respiratory infections can be an ongoing problem for children born prematurely. In particular, they are at increased risk for RSV and influenza. Emerging data from animal research provide insight into mechanisms underlying these findings.

Studies of animals exposed in utero to ethanol suggest that ethanol-induced immune dysfunction persists into adulthood. Specifically, adult animals exposed to ethanol in utero demonstrated impaired adaptive immunity and altered B-cell responses, resulting in increased risk and severity of influenza infection (McGill et al. 2009). Another study (Zhang 2005) demonstrated that in utero ethanol exposure alters the hypothalamic–pituitary–adrenal axis, which in turn results in hyperactivity in stress-induced immunosuppression and increased vulnerability to subsequent infectious illness.

Innate immunity in the lung is impaired in the premature newborn (Bellanti and Zeligs 1995; Hall and Sherman 1992). Growing evidence suggests that in utero ethanol exposure further disrupts multiple arms of innate immunity in the developing lung. Studies in sheep, for example, find that in utero ethanol disrupts immune function by decreasing in the fetal lung surfactant proteins (SP), which also are known as collectins, particularly SP-A and SP-D (Lazic et al. 2007; Sozo et al. 2009). In the lung, these proteins are essential mediators of the local immune response in that they modulate the function of dendritic and T cells and facilitate the removal of pathogens by the alveolar macrophage (Sorenson et al. 2007).

The alveolar macrophage is the resident inflammatory cell that provides the initial defense against foreign and infectious particles and orchestrates the inflammatory process within the lung (Fels and Cohn 1986; Standiford et al. 1995). Alveolar macrophages reside in the lungs’ alveoli and are derived from peripheral circulating blood monocytes (Fels and Cohn 1986; Prieto et al. 1994). As a consequence, anything that affects immune responses of fetal monocytes—for example, exposure to alcohol during pregnancy—may subsequently affect the alveolar macrophage population and the inflammatory environment within the newborn lung (Kramer et al. 2004, 2005).

Furthermore, substances that directly affect alveolar macrophages can therefore affect immunity in the infant lung. Studies in animals find that fetal alcohol exposure decreases the antioxidant glutathione in the fluid lining the alveolar space and within the resident alveolar macrophages (Gauthier et al. 2005*b*). Reductions in glutathione cause oxidative stress in the lung that, in turn, contributes to alveolar macrophage dysfunction and altered alveolar macrophage maturation (Brown et al. 2007; Gauthier et al. 2005*b*, 2010). Other studies in guinea pigs demonstrated that impaired alveolar macrophage function increases the already elevated risk of experimentally induced pneumonia in the newborn pup (Gauthier et al. 2009; Ping et al. 2007). Providing the pregnant guinea pig with the dietary supplement S-adenosylmethionine (SAMe) during ethanol ingestion prevented glutathione depletion in the neonatal lung, protected the neonatal alveolar macrophage from increased reactive oxygen species, improved alveolar macrophage phagocytosis, and decreased the risk of sepsis and pneumonia in the pup. In addition, giving intranasal glutathione treatments to newborn pups exposed in utero to alcohol improved macrophage phagocytosis and diminished lung infections and dissemination of experimentally induced *Group B Streptococcus* pneumonia (Gauthier et al. 2009). These findings support the idea that fetal ethanol exposure causes glutathione depletion in the lung, which in turn decreases the fetal lung’s ability to clear infectious particles and increases the risk of respiratory infections.

Research in both humans and animals suggest that zinc depletion also may play a role in dampening immunity in alcohol exposed infants. Zinc is an essential cofactor in approximately 300 enzyme-dependent processes involved in immunity, growth, cell differentiation, and metabolism (Chandra 2002; Uriu-Adams et al. 2010). Studies of global disease burden for 2010 found that a primary risk factor for death in early infancy was bacterial infection linked to zinc insufficiency (Chaffee and King 2012; Lim et al. 2012; Mori et al. 2012). Indeed, zinc is essential for innate and adaptive immune responses (Knoell and Liu 2010; Maggini et al. 2007), and suboptimal concentrations of zinc result in an increased susceptibility to infection as well as exacerbation of existing infections (Prasad 2013). Newborns are at an increased risk for suboptimal zinc concentrations if their mothers have suboptimal zinc pools, and women who abuse alcohol during pregnancy tend to have suboptimal zinc pools (Keen et al. 2010; Picciano 2003). In addition, researchers have shown that decreases in zinc are a potential relative risk factor for FASD, and zinc supplements may protect against some of the adverse effects of prenatal alcohol exposure (Keen et al. 2010; Picciano 2003). Because approximately 50 percent of pregnancies are unintended (Finer and Henshaw 2006), some mothers may continue drinking during at least part of their pregnancy, resulting in significant fetal alcohol exposure and risk of suboptimal zinc concentrations in newborns. Furthermore, because the majority of zinc is transported across the placenta in the third trimester of pregnancy, newborns born prematurely, before zinc transport is complete, also are zinc deficient (Giles and Doyle 2007), which suggests that premature newborns exposed to alcohol in utero may be at an even higher risk of zinc deficiency.

A study in adult rats suggests a possible mechanism for zinc’s effect on alcohol-induced alveolar macrophage dysfunction. The study found that chronic ethanol ingestion decreased the zinc levels in alveolar macrophage due to decreased expression of zinc transporters (Mehta and Guidot 2012; Mehta et al. 2011). Equally important, dietary zinc restored zinc pools in the alveolar macrophage and improved phagocytosis. Investigations in fetal ethanol models suggest that similar zinc deficiencies contribute to fetal alveolar macrophage dysfunction in the newborn.

## Potential Areas for Further Research

Further research defining the mechanisms underlying alcohol-induced alterations in the immune function of the alcohol-exposed newborn is necessary. In the adult alcohol-exposed lung, alcohol-induced mitochondrial dysfunction significantly contributes to cellular dysfunction and impaired immune response of the alveolar macrophage (Liang et al. 2013, 2014).

Systemically, alcohol alters multiple arms of the immune system. Alcohol-induced increase in intestinal permeability and alterations of the gut microbiome directly contribute to alcohol-associated hepatic inflammation and the progression of liver disease (Chen and Schnabl 2014; Elamin et al. 2013; see also the article by Engen and colleagues). Alcohol-induced changes in gut permeability and the gut’s interaction with the liver modulate both lung and liver inflammation in the setting of burn injury (Chen et al. 2014). Antigen presentation and T-cell dysfunction contribute to the complex immune dysfunction of the alcohol-exposed adult (Fan et al. 2011; Gurung et al. 2009). These important mechanisms have yet to be evaluated among fetuses exposed to alcohol in utero. They remain important potential areas of research particularly in the premature newborn, because morbidities such as late onset sepsis, bronchopulmonary dysplasia, and necrotizing enterocolitis are interrelated (Stoll et al. 2010).

## Conclusion

This article highlights evidence from research in humans and animals suggesting that ingesting alcohol during pregnancy can disrupt the fetal immune system and result in an increased risk of infections and disease in newborns and possibly throughout life. It also emphasizes the critical need for more research to illuminate the strength and nature of this link and the mechanisms by which alcohol may influence the developing immune system.

In particular, researchers need more specific and accurate assays for identifying which newborns have been exposed to alcohol in utero, along with methods to determine the extent and timing of such exposure. Such approaches will allow researchers to determine and more precisely measure the influence of alcohol on infections and diseases related to immune system dysfunction. In addition, continued research is needed to clarify the potential link between alcohol and premature birth, particularly extreme premature delivery.

Evidence from studies in animals has begun to provide theories about how alcohol may disrupt the developing immune system. These animal models already have begun to identify molecular mechanisms in the lung that may directly and indirectly lead to an increased risk of respiratory infections. These studies not only point to potential mechanisms of immune system disruption attributed to in utero alcohol exposure but also to possible interventions that might ameliorate the damage to the developing infant.

## Figures and Tables

**Figure:**
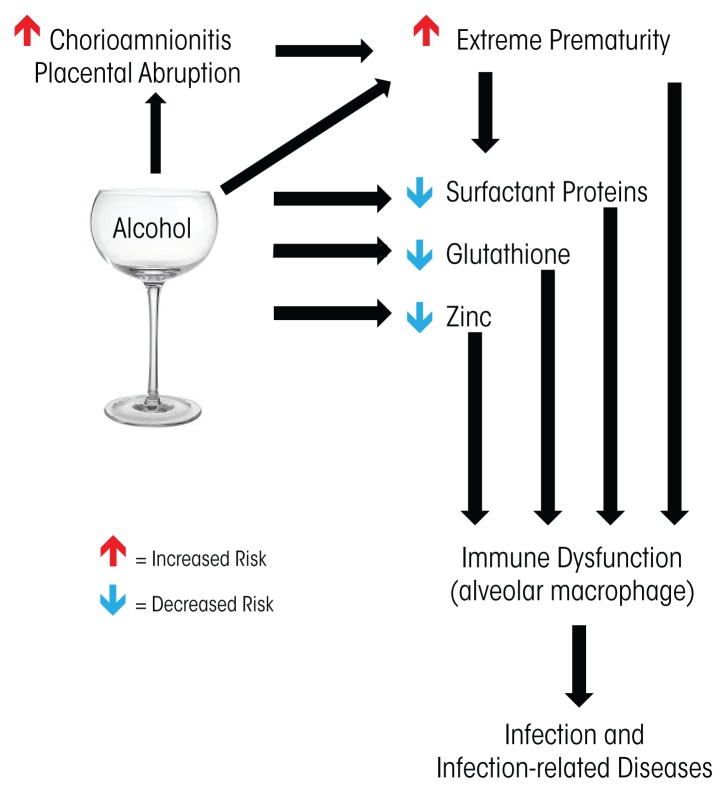
Research suggests that alcohol exposure in utero affects infants’ immune function through a variety of mechanisms, including indirectly by increasing the risk of premature birth and directly by influencing immune mediated defenses, particularly in the lungs.
